# Cardiac function in a large animal model of myocardial infarction at 7 T: deep learning based automatic segmentation increases reproducibility

**DOI:** 10.1038/s41598-024-61417-4

**Published:** 2024-05-14

**Authors:** Alena Kollmann, David Lohr, Markus J. Ankenbrand, Maya Bille, Maxim Terekhov, Michael Hock, Ibrahim Elabyad, Steffen Baltes, Theresa Reiter, Florian Schnitter, Wolfgang R. Bauer, Ulrich Hofmann, Laura M. Schreiber

**Affiliations:** 1 Comprehensive Heart Failure Center (CHFC), Chair of Molecular and Cellular Imaging, University Hospital Würzburg, Würzburg, Germany; 2 Faculty of Biology, Center for Computational and Theoretical Biology (CCTB), University of Würzburg, Würzburg, Germany; 3 Department of Internal Medicine I, University Hospital Würzburg, Würzburg, Germany

**Keywords:** Preclinical research, Heart failure, Magnetic resonance imaging, Data processing, Experimental models of disease

## Abstract

Cardiac magnetic resonance (CMR) imaging allows precise non-invasive quantification of cardiac function. It requires reliable image segmentation for myocardial tissue. Clinically used software usually offers automatic approaches for this step. These are, however, designed for segmentation of human images obtained at clinical field strengths. They reach their limits when applied to preclinical data and ultrahigh field strength (such as CMR of pigs at 7 T). In our study, eleven animals (seven with myocardial infarction) underwent four CMR scans each. Short-axis cine stacks were acquired and used for functional cardiac analysis. End-systolic and end-diastolic images were labelled manually by two observers and inter- and intra-observer variability were assessed. Aiming to make the functional analysis faster and more reproducible, an established deep learning (DL) model for myocardial segmentation in humans was re-trained using our preclinical 7 T data (n = 772 images and labels). We then tested the model on n = 288 images. Excellent agreement in parameters of cardiac function was found between manual and DL segmentation: For ejection fraction (EF) we achieved a Pearson’s *r* of 0.95, an Intraclass correlation coefficient (ICC) of 0.97, and a Coefficient of variability (CoV) of 6.6%. Dice scores were 0.88 for the left ventricle and 0.84 for the myocardium.

## Introduction

Cardiovascular diseases have an immense impact on global public health and are a burden for many people as well as healthcare systems. Not only are they the leading cause of death worldwide, the number of deaths associated with cardiovascular diseases has increased significantly in recent years, amounting to 1.9 million in 2020, equivalent to an increase of 18.7% compared to 2010^1^.

Disease-related changes in cardiac function and morphology can be assessed using cardiac magnetic resonance (CMR) imaging. It has become an increasingly important diagnostic tool which is recommended in the guidelines for a growing number of indications^2,3^ and is considered the gold standard for the quantitative assessment of cardiac function^4,5^.

With regard to CMR, higher field strengths are of growing interest, since they are expected to increase spatial resolution^6^, improving diagnostic value and precision in parameters like cardiac function. In clinical practice, these improvements in precision may enable early disease detection as well as the assessment of small changes in therapy monitoring. In clinical research, the higher precision and therefore more reliable detection of statistical differences directly translates to lower numbers of subjects in a study. This is also very important in preclinical studies, because it reduces the burden on research animals significantly, while simultaneously reducing study costs.

Most 7 T MRI research is done in healthy human subjects. However, many research applications are new and methods still need to be established, making large animal models particularly relevant in contexts where the use of humans would be impractical or unethical. In these cases, animal models allow the testing of specific disease related diagnostics, for example late gadolinium enhancement (LGE) imaging to visualize post-infarction tissue alterations. Pre-clinical studies may thus harvest the benefits of 7 T in a pre-clinical setting before they become available in clinical practice, enabling access to information inaccessible at clinical field strengths. While 7 T CMR imaging has been developing towards clinical applications in humans^7–9^, this process will likely be supported by large animal studies^7–10^.

The assessment of cardiac function based on CMR images requires a precise segmentation of the myocardium. Manual post-processing is not only very time-consuming, but also makes the analysis more subjective and the results less reproducible. Therefore, there are many approaches for fully-automatic segmentation, which are already included in some commercially available software packages used in clinical practice^10^. However, it has been shown that these tools embedded in commercially available clinical software do not perform well in large animal data. They do not provide suitable myocardial segmentation of porcine hearts^11^, so that manual segmentation is needed to calculate cardiac volumes and mass. We encountered the same issues when analysing cardiac cine data from our comprehensive preclinical 7 T CMR study in pigs^12–14^.

The aim of this study was thus to reduce inter- and intra-observer variability of myocardial segmentation in a porcine 7 T CMR study. We used a transfer learning approach for automatic segmentation to increase reproducibility and compared it to manual segmentation. Simultaneously, we aim to demonstrate that an existing deep learning (DL) model already tested regarding 7 T CMR in humans^15^ can be re-trained and adjusted with a relatively small data set and reasonable effort, enabling reliable automatic segmentation of the porcine left ventricle in 7 T CMR images. Sharing our data and models, we aim to provide fully automatic myocardial segmentation to preclinical settings, making cardiac functional analysis faster, more reproducible, and less observer-dependent.

## Methods

The data used in this study are part of a comprehensive large animal study^12–14^. Details of our data can be accessed via the Zenodo repository (see chapter Data availability).

**Figure 1:**
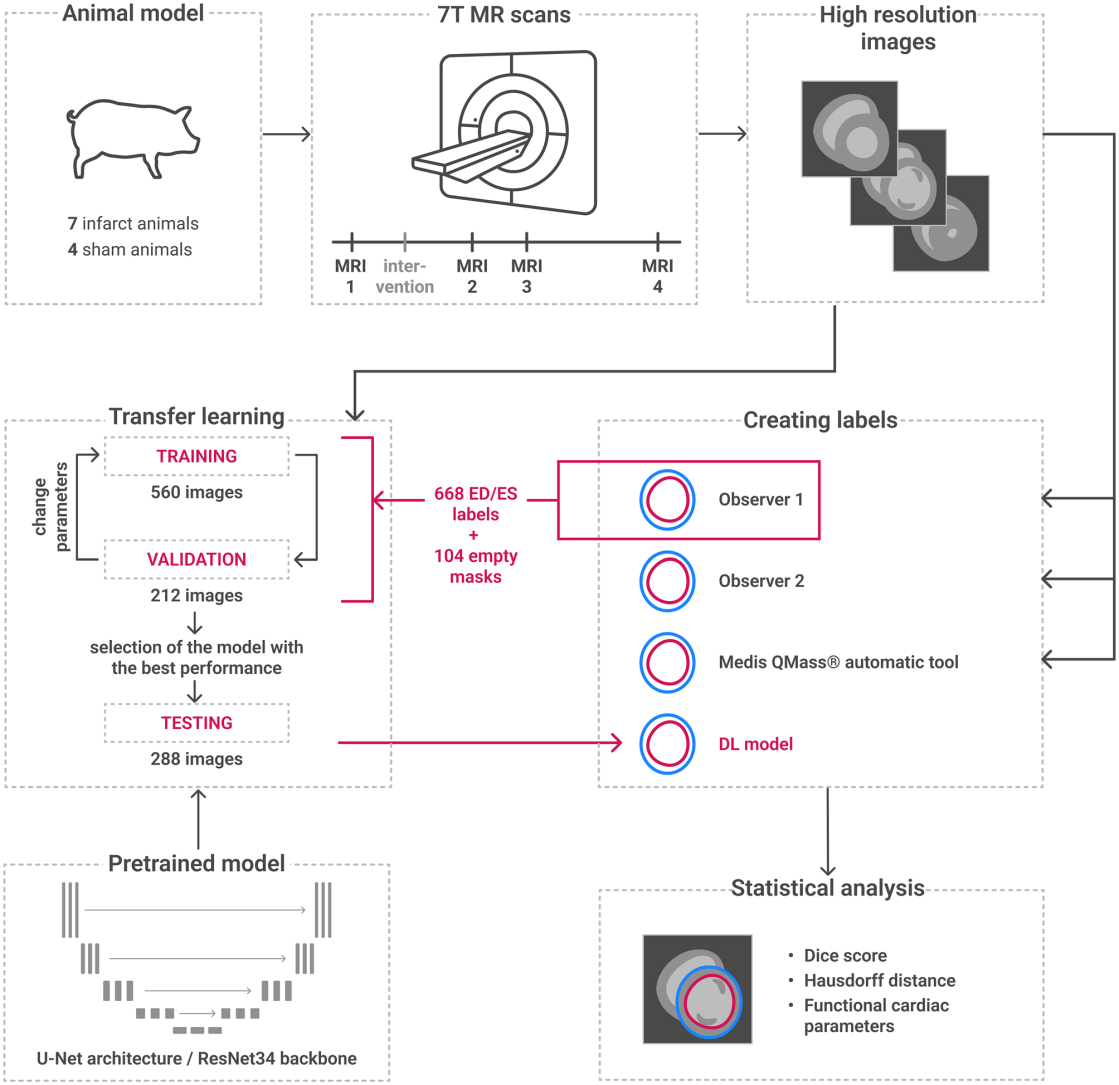
Schematic representation of the design of the study. Eleven animals (seven infarct pigs and four sham pigs) were imaged in a 7 T MR scanner four times each. The acquired high resolution images were labelled using different methods: Two different observers performed a manual segmentation. The end-diastolic and end-systolic labels from observer one were used in a transfer learning algorithm to re-train a DL model. This pre-trained model has a U-Net structure (illustrated schematically) and a ResNet34 backbone. In the transfer learning process, the model was trained using 560 high-resolution 7 T images and the labels of observer one (manually created ED/ES labels or empty segmentation masks if no tissue to be segmented was visible). 212 images served for validation of the model, with which different stages of the model were tested and the performance evaluated. Parameters were changed and the training continued. Then, the model with the best performance (highest dice score) was selected and used as our model. It was tested on 288 images it had not seen so far (test set), and it provided labels for those images. In addition, we segmented the images using an automatic tool within the clinical software Medis^®^. All different segmentations were then compared to each other in a statistical analysis. Dice scores and Hausdorff distances of the labels and the derived cardiac parameters were calculated and compared. *MRI* magnetic resonance imaging, *DL* deep learning, *ED* end-diastolic, *ES* end-systolic.

The methods of image acquisition, DL model training, segmentation, and analysis used in this study are described below. For a schematic illustration of the study procedure, see Fig. 1.

### Animal model

The large animal study was approved by the District Government of Lower Franconia, Germany, (Grant 55.2.2-2532.2-1134-16) and all experiments were performed in accordance with relevant guidelines and regulations. The study report follows recommendations in the ARRIVE guidelines. Details regarding experimental animals and experimental procedures have been previously reported by Schreiber et al.^16^. Experiments were performed in three blocks of n = 4 animals, where the first two blocks belonged to the treatment group and the third block to the sham group. Since one animal died following infarct induction, corresponding data was omitted from this study. No blinding was applied with respect to groups. Blinding applied with respect to outcome and data analysis is described in the section “Manual segmentation”. The same concept was applied to the image quality rating.

We thus included a total number of eleven pigs. In seven of these, myocardial infarction was induced by 90-min occlusion of the left anterior descending artery (LAD) using a balloon catheter inserted via a femoral coronary catheter, after baseline magnetic resonance imaging (MRI).

Four sham animals were used as a control group and received the same intervention with exception of the balloon catheter inflation and occlusion of the coronary artery. Each of the animals underwent a total of four 7 T MRI scans. One baseline scan before the procedure (MRI 1) and three scans (MRI 2–4) at different times (3 ± 1 days, 12 ± 1 days, and 58 ± 1 days) after infarction or sham procedure^12^.

### Cardiovascular magnetic resonance imaging

MR images were acquired on a 7 T MAGNETOM™ Terra system (Siemens Healthineers, Erlangen, Germany). We used three in-house built 8Tx/16Rx coils^17^ of different sizes to adapt to the increasing weight of the pigs throughout the study.

Scan parameters for high-resolution cine imaging were slice thickness: 6 mm, in-plane spatial resolution: 0.4 mm × 0.4 mm, TE/TR: 3.18/49.52 ms, echo spacing: 6.2 ms, bandwidth: 893 Hz/Px and flip angle: optimal (15°–27°). A short-axis stack includes 30 frames per cardiac cycle and 11–16 slices from base to apex. The measurements were performed under breath hold.

### Image quality rating

To assess the quality of the high-resolution cine images, each image in the end diastole and end systole was rated from one (best) to four (worst) based on three criteria (artefacts, noise, and general image quality). The scores were defined as (1) no artefacts/hardly any noise/very good image quality, (2) minor artefacts/noise/reduced image quality that does not affect the delineation of structures, (3) artefacts/noise/reduced image quality that affects the delineation of structures and may lead to misinterpretation, and (4) nondiagnostic image due to major disturbances. The three parameters were then summed up to obtain a total score for each image ranging from three (best possible result) to twelve (worst possible result)^18^.

### Manual segmentation

Post-processing of the obtained MR images was performed using the commercially available software Medis Suite^®^ (QMass^®^, Version 3.1.16.8, Medis Medical Imaging Systems, Leiden, Netherlands).

A standardized procedure was followed for manual segmentation of the short-axis cine stack^19^. The end-systolic and end-diastolic phases were selected based on the visually smallest and largest volume of the left ventricular (LV) blood pool, respectively. Epi- and endocardial borders of the myocardium were then delineated in these phases. Papillary muscles were not excluded from the blood pool, since both in- and exclusion are presented as valid approaches in the guidelines^19^ and the original DL model is not trained to recognize and label papillary muscles.

After one observer completed the segmentation, it was repeated by the same observer after a period of at least one week to evaluate the intra-observer variability. In addition, all scans were segmented by a second observer to assess the inter-observer variability. The two examiners were blinded to each other's segmentation; only the end-diastole and end-systole were set to the same phases for all observers prior to segmentation to allow calculation of Dice scores.

All figures showing CMR images with myocardial contours were processed subsequently. To improve contours with respect to general visibility and colour-blind readers, green and red pixels of the epicardial and endocardial contours were re-coloured blue and magenta, respectively. We used Adobe^®^ Photoshop^®^ CS6 (Version 13.0, Adobe^®^ Systems Incorporated, San Jose, California, USA) for this purpose.

### Commercially available automatic segmentation

CMR analysis software usually provides tools for fully automated LV segmentation. We used Medis Suite^®^ (QMass^®^, Version 3.1.16.8) for CMR post-processing, which is intended for clinical use in human patients. We tested their automatic tool in QMass^®^ on our 7 T images of porcine hearts.

### Deep learning model

Starting point for the deep learning was a pre-trained model published by Ankenbrand et al.^15^ This model has a U-Net architecture^20^ with a ResNet34 backbone^21^ implemented in fastai^22^. Pre-training was performed using cardiac MRI data from the "Data Science Bowl Cardiac Challenge Data”^23^. Prediction is done for three classes (background, left ventricular cavity, and left ventricular myocardium) on images scaled to 256 × 256 pixels.

### Data augmentation

To increase the amount of training data and make the predictions more consistent, various methods of data augmentation were applied. The images were rotated, flipped, and contrast and brightness were changed (flip [left–right], rotation [90°], lighting [0.4] and zoom [1.2]).

### Training process

Scanning the eleven pigs four times each resulted in a total number of 44 scans. Four of those scans had to be excluded from the study as high-resolution short-axis cine stacks were not recorded during the measurements. The remaining forty scans (24 of infarct animals, 16 of sham animals) were divided into three different subsets. This was done animal-wise: six (four infarct and two sham) were assigned to the training set, two (one infarct and one sham) to the validation set and three (two infarct and one sham) to the test set. It was ensured that the animals were divided equally according to infarct or sham group. However, within the groups, the animals were distributed randomly. This resulted in a total of 560 training images, 212 validation images, and 288 images for the test set. Supplementary Table S1 shows the number of images per scan and the division into the subsets for transfer learning in detail.

Re-training of the base model was performed in two steps. In the first step, all parameters except for those from the final parameter group were set as un-trainable (frozen). We trained for 100 epochs this way. An epoch is one full pass through the training data. We used the Adam optimizer^24^ to minimize the general Dice loss as implemented in fastai version 2^22^. At this stage the maximum learning rate which determines how strongly the parameters are adjusted in each optimization step was set to 10^–4^. Checkpoints of the model were saved every 10 epochs. In the second step, models of all 10 checkpoints were compared with respect to the Dice scores on the validation set. The model with the highest Dice score was used as the basis for another 100 epochs with all parameters set as trainable (unfrozen) and maximum learning rate of 10^–5^. Afterwards, the model with the overall highest Dice score on the validation set was selected for further analyses. A test set consisting of scans of three pigs (two infarct pigs and one sham pig, 288 images) was excluded from the training process to evaluate the performance of the model.

### Cardiac magnetic resonance image analysis

The results of the manual segmentation could be calculated directly in QMass^®^, while the contours generated by the DL model had to be imported into the software first. Medis^®^ uses dedicated contour files (.con) to store contour information. DL generated contours were transferred into such a contour file and imported into Medis^®^ for further analysis.

Based on the segmentation, various cardiac parameters were calculated: ejection fraction (EF), stroke volume (SV), LV mass, end-systolic volume (ESV), and end-diastolic volume (EDV). EDV and ESV [ml] were calculated by summing the voxels within the endocardial contour of all slices of the end-diastole and end-systole, respectively. SV [ml] was calculated as EDV minus ESV. EF [%] is expressed as SV divided by EDV, multiplied by 100. LV mass [g] was calculated as the difference of the total epicardial and endocardial volume in end-diastole, multiplied by the specific density of myocardium (1.05 g/ml)^19^.

### Deep learning model performance

The following approach was taken in the overview assessment of the contours generated by the DL model. In some cases, the short-axis stack included images of the base of the heart that were above the part of the heart that guidelines suggest to segment. Therefore, only images that were also labelled manually were included in the evaluation. These were then examined and classified as labelled correctly, incorrectly, or not labelled at all. Any missing or incorrect contours could easily be manually added or adjusted in the software. This was intentionally avoided to be able to compare unedited results.

### Metrics for comparing contours

To quantify how close the automatically generated contours are to the manually drawn contours, we used two geometric metrics^25^:

The Dice score measures the volumetric overlap of two contours, with a value of 1 indicating perfect agreement and 0 indicating no agreement between the two contours. It was calculated for the left ventricle (DICE_LV_) and the myocardium (DICE_MY_). The Dice score of a contour *A* and a contour *B* is calculated as the volumetric overlap of the two contours multiplied by the factor two, and then divided by the two areas of *A* and *B*:

$$Dice\,score=\frac{2\cdot \left|A\cap B\right|}{\left|A\right|+\left|B\right|}.$$

The Hausdorff distance (HD) is the maximum distance between two contours, therefore, a low value indicates high agreement. The HD of two contours *A* and *B* is calculated as follows: The point *a* from contour *A* is determined as the maximum distance to contour *B*. Then, from this point *a*, the minimum distance to a point *b* from contour *B* is determined, resulting in the distance *d*(*a, B*). The same method is used to determine the distance *d*(*b, A*). The HD is now defined as the maximum of these two distances:

$$HD (A,B)=\max \left\{ \underset{a\in A}{\max} d(a,B), \underset{b\in B}{\max}d\left(b,A\right)\right\}$$

with *d*(*a, B*) being the minimal distance from point *a* to contour *B* and *d*(*b, A*) being the minimal distance from point *b* to contour *A*.

Both metrics quantify how strongly the two compared contours agree mathematically.

Images where the DL model provides a label but the observer does not (and vice versa) result in a Dice score of 0 and an infinite HD.

### Statistical methods

We assessed the differences in clinical measures that were calculated based on the two methods of segmentation. All statistical analysis of the predicted cardiac parameters was done using OriginPro^®^, Version 2021 (OriginLab^®^ Corporation, Northampton, Massachusetts, USA) and Microsoft Excel^®^ 2016 (Microsoft®, Redmond, Washington, USA).

Continuous variables were checked for normal distribution using a Shapiro–Wilk test.

Paired Student’s t-tests were performed to test for significant differences. Since for each parameter (EF, SV, LV mass, EDV, and ESV) four hypotheses were tested (observer one vs. repeat, observer one vs. observer two, observer one vs. DL model, and observer one vs. DL model including only scans in the test set), the overall *α* of 0.05 was adjusted according to a Bonferroni correction in order to decrease the risk of a type I error for multiple testing. Therefore, for each t-test a *p*-value of *α* < 0.0125 was considered statistically significant. For the assessment of the intra-class correlation coefficient (ICC), we used a two-way mixed-effects model based on absolute agreement. It was calculated and interpreted according to the guidelines of Koo and Li: Values < 0.5 were classified as poor, between 0.5 and 0.75 as moderate, between 0.75 and 0.9 as good and > 0.9 as excellent^26^. The coefficient of variability (CoV) was calculated as the standard deviation of the difference divided by the mean of two values^27–29^. We used a Bland–Altman analysis to determine intra-observer and inter-observer variability, plotting the difference of the values against the mean of two values^30^. Additionally, Pearson correlation plots were created, and the corresponding *r* values were calculated.

## Results

First, the characteristics of the cohort are reported, then the training process is described, followed by an evaluation of the DL model's performance in comparison to the manual segmentation.

### Characteristics of the cohort

**Figure 2:**
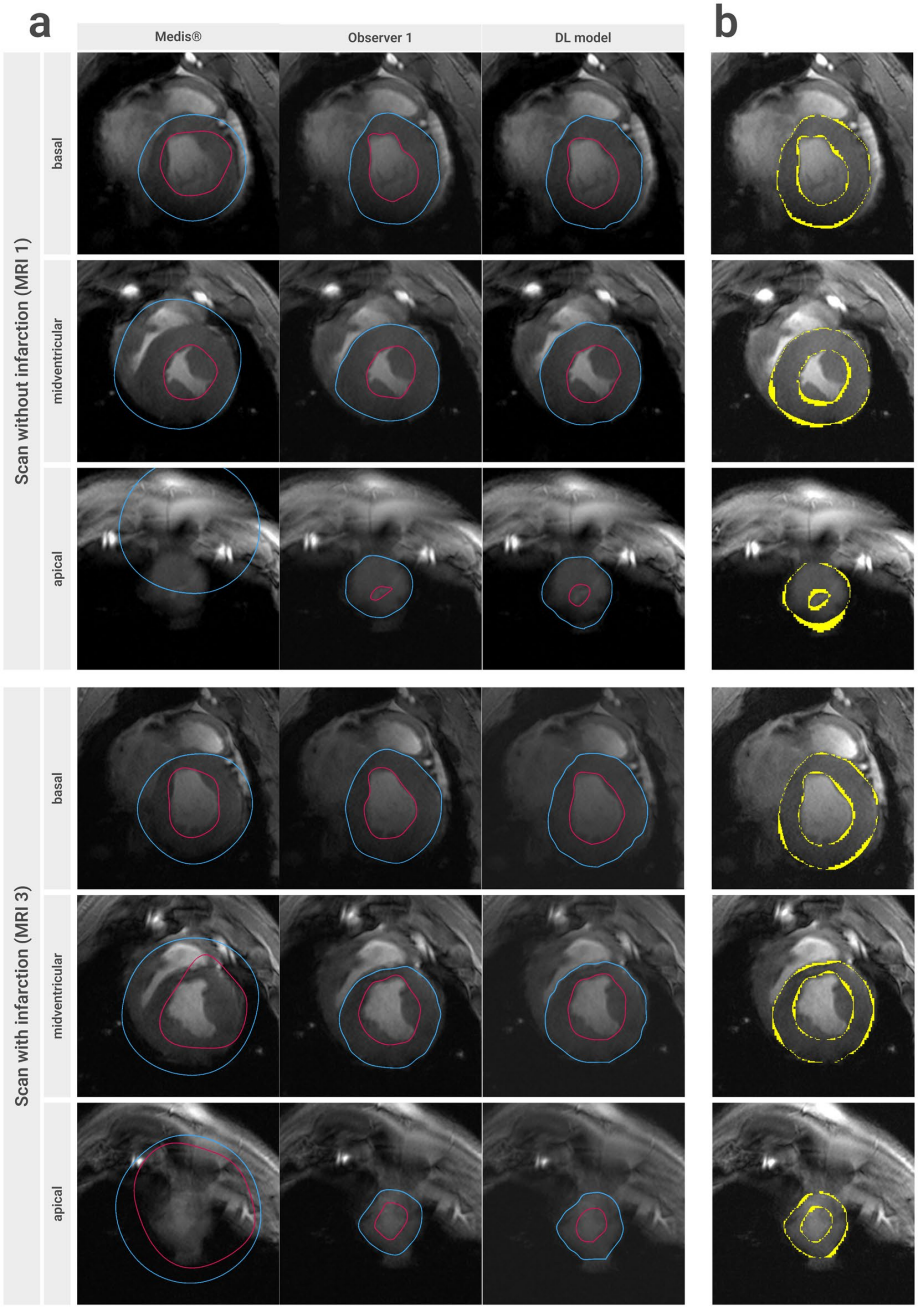
Comparison of different methods of segmentation. (**a**) Comparison of the contours of the automatic segmentation tool in Medis QMass^®^ (left), the manual segmentation of observer one (centre) and the contours created by the DL model (right) in six short-axis cine images. Endocardial contours are drawn in magenta, epicardial contours in blue. A representative single basal, midventricular and apical slice were selected (end-systolic phase). The top images show scans without infarction, the bottom ones show the same heart with subacute infarction (MRI 3, 10 days after infarct procedure). (**b**) Illustration of the differences in the segmentation of observer one and that of the DL model. Pixels that deviate from the ground truth (here: the segmentation of observer one) are highlighted in yellow. *MRI* magnetic resonance imaging, *DL* deep learning.

**Figure 3:**
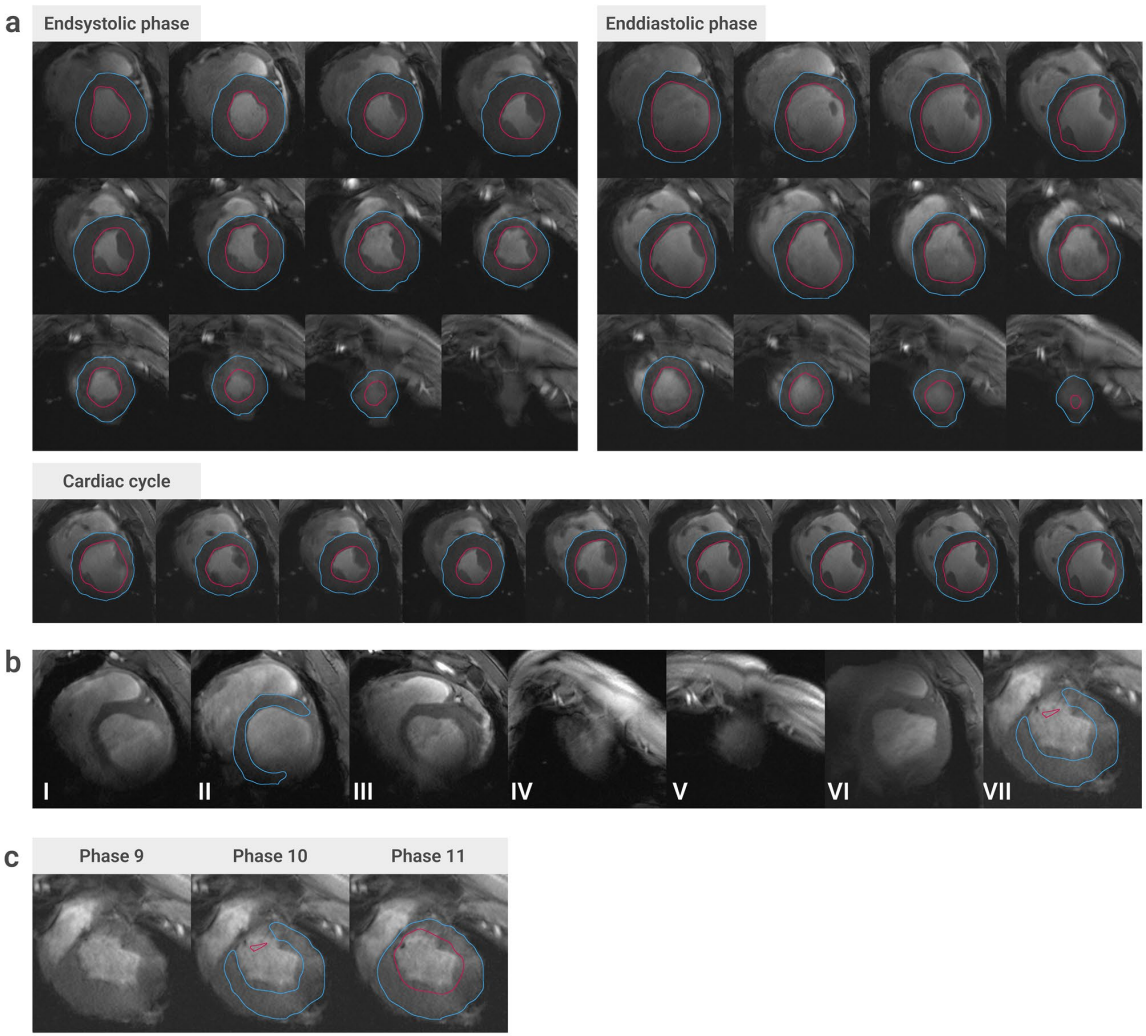
DL model performance. (**a**) DL model segmentation of a short-axis stack of a porcine heart with subacute myocardial infarction (10 days post MI). Endocardial (magenta) and epicardial (blue) contours of the left ventricle are shown in end-diastole (left), end-systole (right) and in a midventricular slice throughout the cardiac cycle (bottom). (**b**) Representative images with missing or incorrect DL prediction. The most likely factors preventing correct segmentation here were: low SNR of the inferolateral wall and low blood-tissue contrast (**I**), low SNR of the inferolateral wall (**II**), artefacts and low SNR of the inferior wall (**III**), artefacts and the lack of a visible blood pool (**IV**), the lack of a visible blood pool (**V**), artefacts in the inferolateral wall (**VI**), and wall thinning in the infarct area (chronic infarction, 59 days post MI), and generally low image quality (**VII**). **(c**) Varying quality of automatic segmentation in three images of adjacent cardiac phases (phase nine to eleven) of a midventricular slice. All images show wall thinning in the infarct area (59 days post MI). In two of them, the image quality and or morphology in this area results in missing and incorrect labels, respectively, whereas in the right image the myocardium was labelled correctly. *DL* deep learning, *SNR* signal to noise ratio, *MI* myocardial infarction.

Figures 2 and 3 show typical 7 T images used for analysis. Results from cardiac function analysis are presented in Table 1.

**Table 1:** Results from manual functional analysis (observer one). The cardiac values are shown for the different scans throughout the study: MRI 1 (baseline scan) and MRI 2–4 at different times after intervention (myocardial infarction or sham procedure, respectively). Values are expressed as mean ± single standard deviation. LV mass is reported in grams, volumes in ml and EF in %. *MRI* magnetic resonance imaging, *EF* ejection fraction, *SV* stroke volume, *LV* left ventricle, *EDV* end-diastolic volume, *ESV* end-systolic volume.

		MRI 1 (baseline scan)	MRI 2 (3 ± 1 days post intervention)	MRI 3 (12 ± 1 days post intervention)	MRI 4 (58 ± 1 days post intervention)
Infarct group	EF [%]	60.6 ± 6.7	40.1 ± 6.8	41.1 ± 5.1	42.2 ± 4.7
	SV [ml]	39.6 ± 8.6	40.6 ± 8.9	42.9 ± 11.8	62.8 ± 8.9
	LV mass [g]	74.8 ± 6.7	94.7 ± 10.3	102.6 ± 8.1	138.6 ± 9.9
	EDV [ml]	65.6 ± 13.5	101.4 ± 15.5	103.0 ± 21.3	149.0 ± 12.9
	ESV [ml]	25.8 ± 7.0	60.6 ± 11.4	60.3 ± 11.8	86.2 ± 9.3
	Weight [kg]	38.8 ± 5.2	42.8 ± 4.6	46.2 ± 4.6	75.8 ± 4.8
Sham group	EF [%]	62.5 ± 2.7	61.5 ± 3.8	59.5 ± 3.4	63.3 ± 4.0
	SV [ml]	28.3 ± 6.2	32.5 ± 3.4	37.3 ± 4.6	62.3 ± 14.0
	LV mass [g]	57.5 ± 11.8	66.0 ± 7.1	69.0 ± 9.5	114.3 ± 17.5
	EDV [ml]	45.0 ± 9.2	52.3 ± 4.3	62.0 ± 6.4	97.0 ± 16.2
	ESV [ml]	16.8 ± 3.3	20.0 ± 2.9	24.8 ± 3.0	35.0 ± 2.1
	Weight [kg]	31.4 ± 6.5	34.7 ± 7.7	37.1 ± 7.1	75.3 ± 9.1

### Image quality rating

The scans were obtained in different series of measurements and have varying image quality. Table 2 shows the ratings for the individual parameters artefacts, noise, and general image quality as well as the overall score. The parameter artefacts was rated 2.6 ± 0.2, noise 1.9 ± 0.3, and general image quality 2.0 ± 0.3, resulting in a total score of 6.5 ± 0.5. The high resolution allowed the recognition of anatomical structures of the heart such as valves, papillary muscles, and trabecular mass.

**Table 2:** Image quality rating sorted by experimental group and sham group. Values are expressed as mean ± single standard deviation. The total score is the sum of values in the three categories artefacts, noise, and general image quality. *n* number of CMR scans in the set.

	Artefacts	Noise	General image quality	Total score
Infarct animal scans (n = 24)	2.6 ± 0.2	2.0 ± 0.3	1.9 ± 0.3	6.6 ± 0.5
Sham animal scans (n = 16)	2.6 ± 0.2	1.8 ± 0.1	2.1 ± 0.2	6.5 ± 0.5
All animals (n = 40)	2.6 ± 0.2	1.9 ± 0.3	2.0 ± 0.3	6.5 ± 0.5

### Commercially available automatic segmentation

The automatic segmentation did not perform properly using the automatic tool within Medis QMass^®^. Representative segmentation results are shown in Fig. 2. Contours were detected in less than 50% of the images, in which in less than 50% the myocardium was correctly identified. In many cases, the epicardial label marked the outer contour of the whole heart. There were particularly severe problems in the basal slices, and in apical slices where no ventricular lumen was present. This is often observed in pigs since their trabeculae are more extensive^31,32^. The correct labelling of epi- and endocardial contours was also impaired by the presence of severe artefacts or a low signal-to-noise ratio (SNR).

### Training process

Throughout the training, the Dice score for all classes increased in the beginning and saturated after 70 epochs of training in the first step (training with frozen parameters). During the second training step (training with unfrozen parameters), the Dice score continued to increase for another 50 epochs before reaching a plateau. Thus, the model selected for further analyses was the one trained for 70 epochs frozen and another 50 epochs unfrozen.

### Performance of the deep learning model

Epi- and endocardial labels were generated by the DL model not only for end-diastole and end-systole, but for all phases. Figure 3a shows an example (MRI 3) where only one end-systolic image was not labelled, while all other depicted images were labelled correctly and visually similar to observer one (see also Fig. 2 for direct comparison). Of all images in the test set that were manually segmented due to guidelines, the model was able to detect and correctly segment the myocardium in 91.8% (3360/3660). In 8.0% (293/3660) of the images in the test set no DL contour was calculated, and contours in another 0.2% (7/3660 images) were not correctly labelling the LV myocardium.

Some visual analysis showed that of those 293 unlabelled images, 123 (equivalent to 42.0%) belonged to the same scan, that of the pig with the lowest body weight (22 kg). Accordingly, the rate of missing contours in this scan was 45.6%, whereas the rate of the remaining ten scans in the test set was 4.6%.

Representative examples of missing or incorrect LV labels are shown in Fig. 3b. Figure 3c shows varying quality of automatic segmentation of the infarcted left ventricle.

### Comparison of results: image segmentation

**Figure 4:**
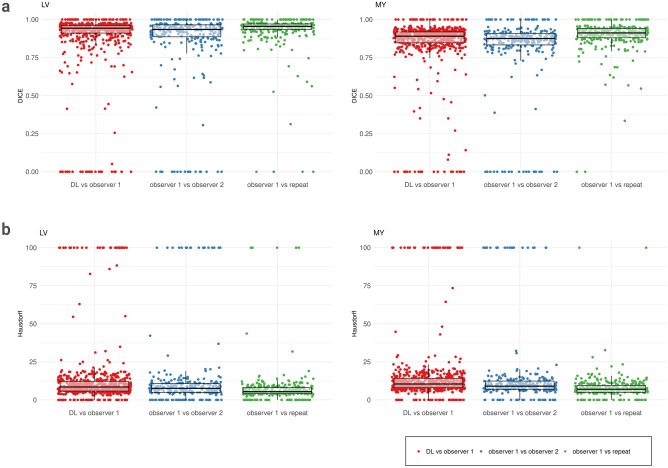
Agreement between segmentation results. (**a**) Dice scores for the left ventricle (left) and the myocardium (right). Each plot contains three different comparisons (from left to right): DL vs. observer one, observer one vs. observer two, and observer one vs. repeat. Each dot represents the Dice score of one image. The box contains all values between the lower and the upper quartile, with the horizontal line representing the median Dice score. (**b**) Hausdorff distances (HD) for the left ventricle (left) and the myocardium (right). Each plot contains three different comparisons (from left to right): DL vs. observer one, observer one vs. observer two, and observer one vs. repeat. Each dot represents the HD of one image. The box contains all values between the lower and the upper quartile, with the horizontal line representing the median HD. All values greater than 100 (including infinite values) were set to 100 for visualisation. *LV* left ventricle, *MY* myocardium, *DL* deep learning.

When comparing the first and second segmentation of observer one (intra-observer analysis), we obtain DICE_MY_ = 0.90, DICE_LV_ = 0.93 and HD_MY_ = 7.0, HD_LV_ = 5.4. For inter-observer analysis between observer one and observer two, we obtain the following values: DICE_MY_ = 0.82, DICE_LV_ = 0.86 and HD_MY_ = 9.0, HD_LV_ = 7.6. When comparing the automatic segmentation of the DL model to the manual delineation of observer one, we receive a mean Dice score of DICE_MY_ = 0.84 (for myocardium) and DICE_LV_ = 0.88 (for the left ventricle). The median HD is HD_MY_ = 10.4 and HD_LV_ = 8.5. See Fig. 4.

### Comparison of results: cardiac function

For results from the cardiac function analysis of the infarction and sham group, see Table 1.

At a significance level *α* = 0.05, the Shapiro–Wilk test classified all obtained values (EF, SV, EDV, ESV, LV mass) of observer one (first and second segmentation), observer two, and the DL model as normally distributed, with exception of the EF of observer one, first segmentation (*p*-value 0.03), the ESV of observer one, both segmentations (*p*-value 0.01 for both), and the ESV of the DL model (*p*-value 0.02). We considered the sample size of n = 40 for the parameters affected to be sufficient to still perform paired Student’s t-tests.

The paired sample t-tests showed significant differences in several cases (Table 3). In the test set, all values obtained using the DL predictions were not significantly different from the values calculated using the manually drawn contours of observer one. Mean differences in cardiac parameters, grouped by the presence of myocardial infarction, are listed in Supplementary Table S2. Overall, values derived from model predictions were closer to the ground truth for animals without myocardial infarction than for those with infarction.

**Table 3:** Mean differences in cardiac parameters and corresponding *p*-values for paired Student’s t-tests. At an *α*-level of 0.0125 statistically significant differences are highlighted by bold font for metrics and corresponding *p*-values. *EF* ejection fraction, *SV* stroke volume, *LV* left ventricle, *EDV* end-diastolic volume, *ESV* end-systolic volume, *DL* deep learning, *n* number of scans included in the comparison.

	Observer one vs. repeat (n = 40)	Observer one vs. observer two (n = 40)	Observer one vs. DL model (all scans, n = 40)	Observer one vs. DL model (scans in the test set, n = 11)
ΔEF [%]	**0.88 (*p* = 0.005)**	**5.08 (*p* < 0.001)**	1.45 (*p* = 0.041)	1.55 (*p* = 0.353)
ΔSV [ml]	< 0.001 (*p* = 1.000)	**2.80 (*p* < 0.001)**	**2.35 (*p* = 0.008)**	2.82 (*p* = 0.266)
ΔLV mass [g]	0.13 (*p* = 0.885)	3.48 (*p* = 0.028)	**− 7.7 (*p* < 0.001)**	**− **9.36 (*p* = 0.029)
ΔEDV [ml]	**− 1.55 (*p* = 0.002)**	**− 2.30 (*p* = 0.011)**	2.60 (*p* = 0.016)	3.82 (*p* = 0.243)
ΔESV [ml]	**− 1.48 (*p* < 0.001)**	**− 5.35 (*p* < 0.001)**	0.25 (*p* = 0.710)	0.91 (*p* = 0.629)

CoV and ICC values measuring inter- and intra-observer reproducibility and corresponding literature values for the CoV are displayed in Table 4. In the clinical context, the CoV is usually calculated to measure inter-observer variability, while publications on deep learning tend to use the Dice score or the ICC. Literature values for the ICC are given in the discussion to evaluate the DL model performance. The CoV for observer one vs. DL model (test set) ranged from 6.6 to 11.3% with a mean value of 8.4% (not displayed in Table 4).

**Table 4:** Intra- and inter-observer reproducibility: CoVs and ICCs for different parameters of cardiac function. Referenced literature values for intra- and inter-observer reproducibility are given below for each of the two coefficients. Values are mean values of all scans (n = 40). For the ICC we used a two-way mixed-effects model based on absolute agreement. *CoV* Coefficient of variability, *ICC* Intra-class correlation coefficient, *EF* ejection fraction, *SV* stroke volume, *LV* left ventricle, *EDV* end-diastolic volume, *ESV* end-systolic volume.

		EF	SV	LV mass	EDV	ESV
Intra-observer (observer one vs. repeat)	ICC	0.99	0.99	0.99	0.99	0.99
	CoV	2.4%	3.4%	3.6%	2.3%	3.3%
	Literature values—CoV	0.01–9.8%^11,29,33–37^	2.6–17.2%^29,35–37^	3.3–15.4%^11,29,33–37^	2.1–14.3%^29,33–37^	5.8–18.8%^29,33,35–37^
Inter-observer (observer one vs. observer two)	ICC	0.90	0.97	0.96	0.99	0.98
	CoV	8.0%	8.5%	7.7%	3.7%	8.7%
	Literature values—CoV	2.3–9.5%^29,33–38^	3.3–12.5%^29,35–38^	3.7–12.9%^29,33–38^	2.6–18.7%^29,33–38^	6.8–16.7%^29,33,35–38^
Inter-observer (observer one vs. DL model)	ICC	0.97	0.96	0.96	0.99	0.99
	CoV	6.0%	9.6%	6.0%	5.6%	6.5%
	Literature values—CoV	2.3–9.5%^29,33–38^	3.3–12.5%^29,35–38^	3.7–12.9%^29,33–38^	2.6–18.7%^29,33–38^	6.8–16.7%^29,33,35–38^

Due to consistent predictions, a CoV of 0% and an ICC = 1 are received for intra-observer variability of the DL model.

**Figure 5:**
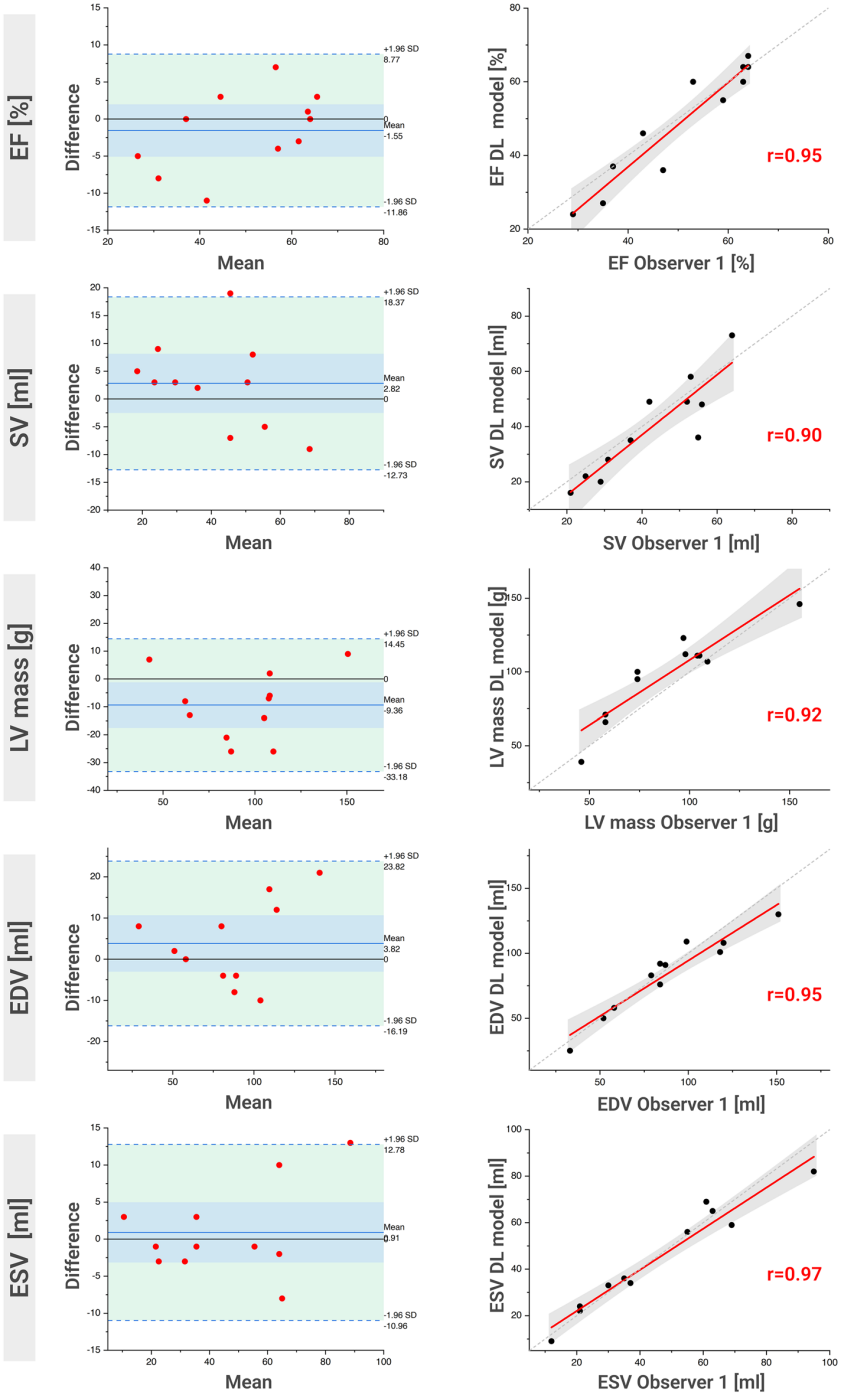
Agreement of LV volumes and mass between observer one and DL model. The left column shows Bland–Altman plots for EF, SV, LV mass, EDV and ESV calculated based on the test set (a set of images that was not used for training or validation of the DL model). In each plot, the horizontal blue line shows the mean difference, and the light green area represents the range between ± 1.96 standard deviations of the differences. The column on the right shows Pearson correlation plots for EF, SV, LV mass, EDV and ESV, again using only scans from the test set. Each one plots the value of observer one (x-axis) against the value of the DL model (y-axis). The continuous red line represents the linear fit of the values, the dashed grey line the bisector (*f*(*x*) = *x*), and the grey area the confidence band (95% confidence interval). The corresponding Pearson’s *r* values are given in red. *EF* ejection fraction, *SV* stroke volume, *LV* left ventricle, *EDV* end-diastolic volume, *ESV* end-systolic volume, *DL* deep learning.

Figure 5 displays Bland–Altman plots for metrics of cardiac function derived from observer one and the DL model. With a few exceptions, all values lie within ± 1.96 standard deviations. In some plots, there is a systematic deviation of the mean difference from *y* = 0 between observer one and the DL model. This can be observed particularly for SV and LV mass, where the value of the DL model tends to be lower and higher, respectively, than that of observer one.

*R* values are displayed as Pearson correlation plots (see also Fig. 5). The intra-observer comparison (observer one) shows a mean value of *r* = 0.99, observer one compared to observer two has a mean value of *r* = 0.95. The values for observer one vs. DL model range between *r* = 0.90 and *r* = 0.97, with a mean value of *r* = 0.94. When including all scans (not shown), they increase to values between *r* = 0.94 and *r* = 0.99, mean value *r* = 0.96.

## Discussion

The present study is to our knowledge the first approach to fully automatic myocardial segmentation in large animals. We present a well performing DL model for automatic LV segmentation in 7 T images of healthy and infarcted (acute to chronic) porcine hearts.

Due to the animal model and the longitudinal study protocol, training images for DL model were sourced from a heterogeneous group of both healthy and diseased animals. Furthermore, we observed some variance in image quality (artefacts and noise as described previously). While this may be considered as a disadvantage in a clinical setting, we considered it to be an advantage in this particular study, where our aim was to train a well performing and generalizing DL model that can be applied in future CMR large animal studies.

The heterogeneity in image quality was due to various factors such as the use of different coils to accommodate growth of the pigs, animals of different disease states (healthy to chronically infarcted hearts), and the varying quality of ECG/acoustic gating during the scans, which was for example impaired by post-infarction arrhythmias. Additionally, we observed B_0_ inhomogeneity and resulting susceptibility artefacts, which were present mainly at the posterior wall and caused by the interface between myocardial and lung tissue, and B_1_ inhomogeneity and resulting signal voids or signal variations. While B_1_ inhomogeneity was addressed quite well with the use of three different RF-coils in this study, B_0_ and B_1_ inhomogeneity remain challenging issues in 7 T CMR.

As mentioned above, we considered the varying heart morphology and image quality as advantageous and therefore chose to use all of the acquired images for transfer learning, not just those of good or optimal quality. This also allowed us to test the DL model under difficult conditions for myocardial segmentation. Since it has already been shown for humans that it is sufficient to train a DL model with end-diastolic and end-systolic labels only^15^, we followed this approach that significantly reduces the required time for label generation, but also for model training and validation.

Initially, considering their development mainly for human use at clinical field strength, it was unclear if a commercial image segmentation tool would be usable in our study. Similar to another research group mentioned earlier^11^, we found that the tested commercial software tool could not be directly applied to preclinical data. Our study demonstrated that the use of commercial software currently may need further checks and adaptations before being used in pipelines for preclinical 7 T CMR image analysis. At this point it has to be mentioned that the automatic tool of only one post-processing software was tested and thus no statement about the performance of other software packages is possible.

Overall, high Dice scores and low HDs for model predictions of both epicardial and endocardial contours indicated high segmentation agreement with the two human observers.

For individual end-diastolic and end-systolic images, the DL model did not provide a segmentation label. As pointed out in the results, the rate of missing labels was particularly high for one scan (45.6% missing labels) compared to the other scans (4.6% missing labels). This scan contained data from the baseline (prior to MI) measurements of the lightest pig of this study (22 kg body weight). The mean body weight of the other animals for the baseline was (35.5 ± 6.9) kg. The pig’s small body and heart size introduced difficulties with respect to cardiac planning. Following standard procedures did not result in proper short-axis orientation, but rather a pseudo short-axis orientation that could not be resolved despite multiple attempts. The resulting atypical left ventricular morphology (compare Supplementary Fig. S1) deviates from all other typical short-axis images in the study. We consider this to be the reason for the high rate of missing labels in this scan.

Note that in the images with missing or incorrect labels, the factors mentioned such as artefacts, low SNR, or problems in the infarct area (see Fig. 3b for examples), did not always prevent correct segmentation. Over 90% of the images were segmented correctly, whereas, for instance, B_0_ artefacts were present in many images, since those are very common when performing measurements at 7 T. The presence of infarct-typical morphology was also not rare, since about 50% of the images come from pigs with myocardial infarction. It is not possible to state exactly what the decisive factor is that prevents correct segmentation in each case. It is often observed that images are not segmented or segmented incorrectly, although images from adjacent cardiac phases, which differ only slightly, are labelled correctly. An example is shown in Fig. 3c, where we considered the infarct area to be the cause of the incorrect (phase 10) or missing label (phase 9). Although there is only a minimal visual difference, the infarcted myocardium was labelled correctly in phase 11, as it was in most images with infarction. While further analysis was beyond the scope of this study, such information could be gained via attention mapping, where areas of images are mapped based on their impact on the model decision, essentially visualizing the attention of the model to different image regions^39,40^.

Values for inter- and intra-observer variability assessed as CoV and ICC as well as literature values for comparison are listed in Table 4^29,33–38,41,42^. Since this is the first study to analyse inter- and intra-observer reproducibility in a large animal model at 7 T, no directly comparable studies were available. The studies referenced used clinical field strengths (1.5 T and 3 T), were mostly analysing human hearts, and followed different approaches concerning the in- or exclusion of papillary muscles. Especially for the intra-observer comparison, the calculated CoV is excellent and at the lower end of the reference range, indicating that the achieved reproducibility in metrics of cardiac function in this study can be considered comparatively high. All ICC values were > 0.9 and therefore interpreted as “excellent”.

The literature above is focussed on manual segmentation. Regarding the evaluation of DL models, only limited literature reports exist that focus on the accuracy of metrics of cardiac function, since they rather use segmentation metrics such as the DICE coefficient or the HD. For the CoV of the EF, Backhaus et al.^37^ received values of 6.5% and 6.7%, respectively, depending on if the automatically generated values were compared to those of an experienced or an inexperienced human observer. Schuster et al.^41^ received a CoV of 12.3%. Regarding EF, our calculated CoV was 6.6% (test set) and 6.0% (scans of all sets). For LV mass, Backhaus et al.^37^ found a CoV of 8.7% and 18.7%, respectively, and Schuster et al.^41^ one of 14.2%. Our CoV for LV mass was 8.4% (test set) and 6.0% (scans of all sets). For both cardiac parameters, our CoVs are comparable to or lower than what has been reported.

ICC values (DL model vs. observer one, test-set) were comparable to what has been found in other previously mentioned automatic segmentation studies: Our ICC for EF was 0.97, while the referenced studies found ICCs of 0.88 and 0.97^37,41^. Regarding LV mass, we found an ICC of 0.94, while reference ICCs ranged between 0.84 and 0.99^37,41^. Also with regard to the ICCs, the results of our model indicate a comparatively high inter-observer reproducibility.

Thus, within our study, obtained parameters of cardiac function show overall good agreement between DL model and human observer and metrics evaluating reproducibility are consistent with or improved compared to literature reports at clinical field strengths.

When directly comparing the resulting parameters of cardiac function, the mean differences between the DL model and observer one are overall comparable to the mean differences between the two human observers (see Table 3). Paired Student’s t-tests comparing the values of the DL model and observer one (including the values of all scans) showed significant differences for the parameters SV and LV mass. When including only scans in the test set, no significant differences between the values of the DL model and those of observer one were found. Mean differences in the test set comparison were higher than in the all scans comparison. The paired t-tests showing no significant differences in the test set comparison should thus be considered a result of the smaller sample size in the test set (n = 11) rather than a sign of better agreement and model performance.

Dividing the animals by the presence or absence of myocardial infarction showed overall higher mean differences between model predictions and ground truth in the infarction group (see Supplementary Table S2). The higher deviation in EDV and the derived metrics (SV and EF) in the infarct group may be related to less consistent heart rates during image acquisition. However, due to the small sample sizes (n = 5 and n = 6) in the test set comparison, the DL model’s performance on individual scans can have a major impact on the mean difference in this comparison of subgroups, restricting generalized conclusions.

As mentioned, the DL segmentations were not edited subsequently. Incorrect contours were not corrected and missing contours were not added manually. In some cases, contours for the end-systolic or end-diastolic phase were missing. This artificially reduced the volume in end-systole or end-diastole (ESV and EDV), which consecutively affected the calculation of the EF.

In the Bland–Altman plots visualising the agreement of observer one and the DL model (see Fig. 5), a systematic underestimation of the SV by 2.35 ml and an overestimation of the LV mass by 7.7 g is observed. Compared to the average values of all scans of all animals, this corresponds to a deviation of 5.4% (2.35 ml deviation with a mean value of 43.4 ml) and 8.4% (7.7 g deviation with a mean value of 91.9 g), respectively. A tendency of the model to draw the endocardial contour more inward is apparent, which increases the LV myocardial mass but decreases the LV volume (especially in end-diastole). We attribute this mainly to the fact that a clear discrimination between myocardium and papillary muscle is sometimes very difficult or even impossible, especially in apical slices. Pigs are well suited for cardiac studies as their heart anatomy largely corresponds to that of humans^31,43,44^. One difference, however, is that the papillary muscles are more extensive^31,32^, which often results in drastically reduced lumen and therefore blood pool in apical slices. Drawing anatomically correct endocardial contours in these slices is already challenging for human observers, leading to differences from scan to scan. Such inconsistencies are introduced in the model training as well, indicating trends and biases in the training data that are not necessarily perceivable by visual inspection. The advantage of an automatic segmentation model is that the decision on how to draw the endocardial contour in such a case is made consistently. As pointed out above, a human observer, on the other hand, will probably draw the contour sometimes more conservatively and sometimes less conservatively over time.

The good agreement between the DL model segmentation and manual segmentation raises the question of whether there are cases where the model outperforms a human observer. Intra-observer variability is typically smaller than inter-observer variability, as can be observed in Table 3. For all cardiac parameters, the difference between two observers is greater than that between two repeated measurements of the same observer. One important finding is that for the parameters EF, SV, and ESV, the difference between the model and observer one is smaller than that of the two human observers. Especially for the diagnostically relevant parameter EF, the model's values are substantially closer to observer one than those of the second observer are.

Together with the fact that once the training is finished, a model makes consistent predictions corresponding to a CoV of 0% and an ICC of 1, this highlights the benefits of using DL, particularly in preclinical research. Here, higher reproducibility in analysis directly translates to a lower number of animals required for a study. The possibility of limiting the number of animals in a study (reduction) is crucial considering the previously described fact that for some applications in ultra-high field MRI animals may be irreplaceable (replacement as another of the 3R principles^45^ of laboratory animal protection). This emphasizes the importance of high reproducibility in image analysis in preclinical research, making it an ideal setting for the application of DL models.

There is currently no commercial tool that provides reliable automatic segmentation of the left ventricle for large animals. Therefore, to benefit from automatic segmentation, one needs to train a model for this respective case. We have demonstrated that it is feasible to train such a model for automatic segmentation of 7 T CMR images of porcine hearts with reasonable effort using transfer learning. It is noteworthy that transfer learning can induce an effect called catastrophic forgetting^46^, where the adaptation of weights due to training with new data or new target tasks can negatively impact the performance of the model on the original data or task. Careful consideration of the target data is therefore required to profit from transfer learning. By publishing this study in combination with data and code, we hope to encourage other groups that analyse pre-clinical CMR images and have not yet been able to use clinical software tools for automatic segmentation to use our approach.

### Supplementary Information

Supplementary Information.

## Data Availability

The datasets generated and analysed during the current study are available in the Zenodo repository [https://doi.org/10.5281/zenodo.7684034]. The source code is available in the GitHub repository [https://github.com/chfc-cmi/cmr-seg-tl-sscrofa].
