# Supplementary material for: Cardiac function in a large animal model of myocardial infarction at 7 T: deep learning based automatic segmentation increases reproducibility
Source: Sci Rep. 2024 May 14;14:11009. doi: 10.1038/s41598-024-61417-4 (PMC11094053; doi:10.1038/s41598-024-61417-4)
Supplement: Supplementary file 1 — Supplementary Information. [file 41598_2024_61417_MOESM1_ESM.docx]

**– SUPPLEMENTARY MATERIAL –**

**Cardiac Function in a Large Animal Model of Myocardial Infarction at 7T -
Deep Learning Based Automatic Segmentation Increases Reproducibility**

Alena Kollmann^1*^, David Lohr^1*^, Markus J. Ankenbrand^2^, Maya Bille^1^, Maxim Terekhov^1^, Michael Hock^1^, Ibrahim Elabyad^1^, Steffen Baltes^1^, Theresa Reiter^1,3^, Florian Schnitter^3^, Wolfgang R. Bauer^3^, Ulrich Hofmann^3^, and Laura M. Schreiber^1^

^1^University Hospital Würzburg, Comprehensive Heart Failure Center (CHFC), Chair of Molecular and Cellular Imaging, Würzburg, Germany

^2^University of Würzburg, Faculty of Biology, Center for Computational and Theoretical Biology (CCTB), Würzburg, Germany

^3^University Hospital Würzburg, Department of Internal Medicine I, Würzburg, Germany

*contributed equally

Corresponding author:

Dr. rer. nat. David Lohr

University Hospital Würzburg, Comprehensive Heart Failure Center (CHFC)

Chair of Molecular and Cellular Imaging

Am Schwarzenberg 15, 97078 Würzburg

Correspondence E-mail: Schreiber_L@ukw.de

Parts of this paper will be used in the doctoral thesis of A. Kollmann.

Planned journal: Nature scientific reports

**Supplementary tables**

**Supplementary Table S1.** Distribution of animals and scans within the training, validation, and test set. The table shows the number of images per scan (ED and ES images). Pig D was excluded from the study, since it died during infarct induction. Four scans had to be excluded from the study as high-resolution short-axis cine stacks were not recorded. The number of images per scan depends on the number of slices, which is proportional to the heart size. The increase in heart size over the study duration thus results in a larger number of images for MRI 4 compared to MRI 1. In total, the training set consisted of 560, the validation set of 212, and the test set of 288 images.

MRI: magnetic resonance imaging, ED: end-diastolic, ES: end-systolic.

|  | **Pig** | **MRI 1** | **MRI 2** | **MRI 3** | **MRI 4** | **Set** |  |
| --- | --- | --- | --- | --- | --- | --- | --- |
| **Infarct animals** | Pig A | excluded | 22 | 24 | 32 | Training |  |
|  | Pig B | excluded | 22 | 26 | excluded | Training |  |
|  | Pig C | 24 | 26 | 26 | 24 | Test |  |
|  | Pig E | 26 | 28 | 26 | 26 | Validation |  |
|  | Pig F | 26 | 26 | 28 | 32 | Training |  |
|  | Pig G | 26 | 26 | 28 | 28 | Training |  |
|  | Pig H | 28 | 28 | 28 | excluded | Test |  |
| **Sham animals** | Pig I | 26 | 24 | 26 | 28 | Test |  |
|  | Pig J | 24 | 26 | 26 | 30 | Validation |  |
|  | Pig K | 24 | 26 | 26 | 28 | Training |  |
|  | Pig L | 28 | 28 | 26 | 28 | Training |  |

**Supplementary Table S2.** Mean differences in cardiac parameters, grouped by the presence or absence of myocardial infarction. The upper half of the table contains data from all scans of animals with infarction (animals in the infarct group: MRI 2, 3, and 4). The bottom half contains data from all scans of animals without myocardial infarction (animals in the infarct group: MRI 1 as baseline scan before infarct induction and animals in the sham group: all). The paired t-tests were not repeated for the subgroups, since the very small sample size and α-level adaptations for multiple testing would provide very limited statistical power.

MI: myocardial infarction, EF: ejection fraction, SV: stroke volume, LV: left ventricle, EDV: end-diastolic volume, ESV: end-systolic volume, DL: deep learning, n: number of scans included in the comparison.

| **Animals** **with MI**  (Infarct group, MRI 2, 3 and 4) | **Observer one**  **vs. repeat**  (n = 19) | **Observer one**  **vs. observer two** (n = 19) | **Observer one**  **vs. DL model**  (all scans, n = 19) | **Observer one**  **vs. DL model**  (scans in the test set, n = 5) |
| --- | --- | --- | --- | --- |
| **ΔEF** [%] | 1.00 | 3.21 | 2.63 | 4.20 |
| **ΔSV** [ml] | 1.21 | 2.47 | 4.68 | 8.40 |
| **ΔLV mass** [g] | -0.21 | 4.21 | -5.53 | -3.20 |
| **ΔEDV** [ml] | 0.26 | -1.89 | 5.68 | 10.80 |
| **ΔESV** [ml] | -0.89 | -5.00 | 1.00 | 2.40 |
| **Animals** **without MI**  (Infarct group, MRI 1 and sham group) | **Observer one**  **vs. repeat**  (n = 21) | **Observer one**  **vs. observer two** (n = 21) | **Observer one**  **vs. DL model**  (all scans, n = 21) | **Observer one**  **vs. DL model**  (scans in the test set, n = 6) |
| **ΔEF** [%] | 0.76 | 6.76 | 0.38 | -0.67 |
| **ΔSV** [ml] | -1.10 | 3.10 | 0.24 | -1.83 |
| **ΔLV mass** [g] | 0.43 | 2.81 | -9.67 | -14.50 |
| **ΔEDV** [ml] | -3.19 | -2.67 | -0.19 | -2.00 |
| **ΔESV** [ml] | -2.00 | -5.67 | -0.43 | -0.33 |

**Supplementary figure**


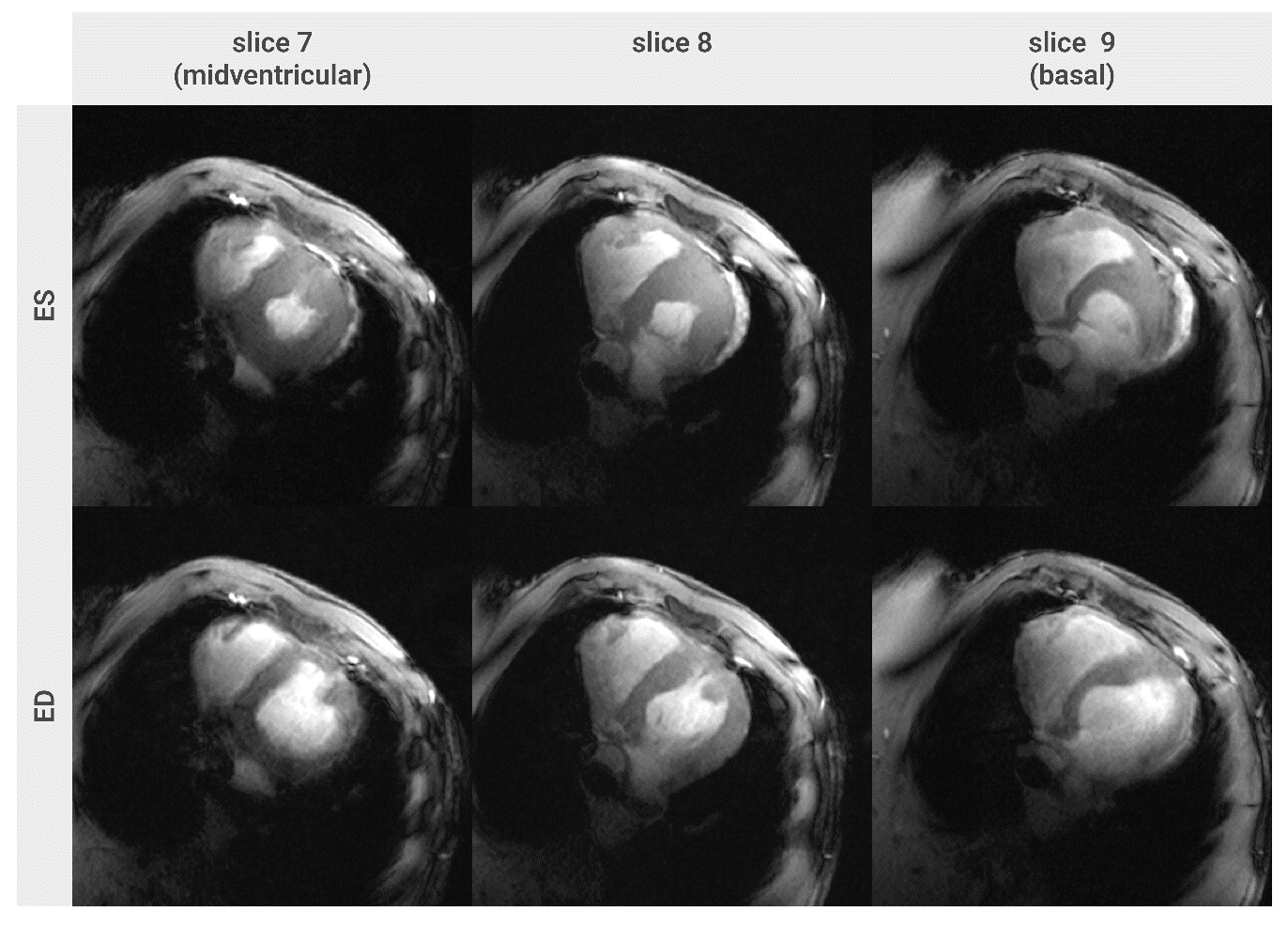
**Supplementary Figure S1.** Basal and midventricular images in end-systole (top row) and end-diastole (bottom row), illustrating the atypical left ventricular morphology of the lightest pig scanned (baseline scan, MRI 1). The pig’s small body and heart size caused difficulties with respect to cardiac planning, resulting in a pseudo short-axis orientation. The atypical morphology with the left ventricle appearing in an incomplete circumferential configuration (particularly in basal slices) was considered the reason for the high rate of missing DL model-predicted labels in this scan.

ED: end-diastole, ES: end-systole.
